# A vectorial tree distance measure

**DOI:** 10.1038/s41598-022-08360-4

**Published:** 2022-03-28

**Authors:** Avner Priel, Boaz Tamir

**Affiliations:** 1 The Mina & Everard Goodman Faculty of Life Sciences, Bar Ilan University, Ramat Gan, Israel; 2 Faculty of Engineering, Tel-Aviv University, Tel Aviv, Israel; 3 Faculty of Interdisciplinary Studies, Bar Ilan University, Ramat Gan, Israel

**Keywords:** Computational biology and bioinformatics, Developmental biology

## Abstract

A vectorial distance measure for trees is presented. Given two trees, we define a Tree-Alignment (T-Alignment). We T-align the trees from their centers outwards, starting from the root-branches, to make the next level as similar as possible. The algorithm is recursive; condition on the T-alignment of the root-branches we T-align the sub-branches, thereafter each T-alignment is conditioned on the previous one. We define a minimal T-alignment under a lexicographic order which follows the intuition that the differences between the two trees constitutes a vector. Given such a minimal T-alignment, the difference in the number of branches calculated at any level defines the entry of the distance vector at that level. We compare our algorithm to other well-known tree distance measures in the task of clustering sets of phylogenetic trees. We use the TreeSimGM simulator for generating stochastic phylogenetic trees. The vectorial tree distance (VTD) can successfully separate symmetric from asymmetric trees, and hierarchical from non-hierarchical trees. We also test the algorithm as a classifier of phylogenetic trees extracted from two members of the fungi kingdom, mushrooms and mildews, thus showimg that the algorithm can separate real world phylogenetic trees. The Matlab code can be accessed via: https://gitlab.com/avner.priel/vectorial-tree-distance.

## Introduction

A distance measure between two trees can be calculated based on several approaches. One can compare the two adjacency matrices by evaluating the eigenvalues or eigenvalue gaps^1^. Alternatively one can compare graph properties, such as centrality, density, etc. A well known distance measure is the Tree Edit Distance (TED)^2–4^. In the TED we look for the optimal set of editing actions (e.g., insertion, deletion) transforming one tree into the other. One can cut any of the two trees at any level, where an edge can be inserted. There are several distance measures for phylogenetic trees. Each phylogenetic tree comes with a set of labels (taxa) attributed to the leaves. In the Robinson–Foulds measure^5^ two operations are defined, a contraction where an edge is deleted and the labels on the leaves are rearranged, and an inverse operation, a de-contraction where an edge is added, and a corresponding new partition of the labels is set. The minimal sequence of such operations leading one tree into the other defines the distance measure. In^6^ a tanglegram is defined by comparing the two sets of labels, counting the minimal number of crossings of lines joining the corresponding labels. In^7^ a score was computed to each pair of edges, based on the partition of labels defined by each edge, next an alignment of the trees was calculated to maximize the sum of scores. Recently, several software tools for the presentation of trees were suggested^8^.

Another approach was suggested more recently, where one uses machine learning kernel methods to compare two trees. In kernel methods^9,10^ one maps the original data space into some feature space to compare two trees by computing their scalar ‘dot product’ . The feature space could be of high dimension, however we can use the ‘kernel trick’^11^ to compute such dot products. Two trees are considered similar if their normalized scalar product in the feature space is close to 1, and orthogonal or different if this product is 0. The question remains: which feature space is appropriate for the task? There are several families of known feature spaces for trees, making the following list of kernel methods:

- Convolutional Kernels, where kernels on the tree set are induced from kernels on subsets, such as subtrees^12^ or subset trees^13,14^. Similarly in^15^ an approximate tree kernel was discussed in the context of NLP. Partial tree kernel were suggested by^16^. Elastic tree kernels were discussed in^17^, Grammar Driven tree kernels^18^, Semantic-Syntactic tree kernels in^19^. All of the above methods, being applied by a mapping into some feature space, lose some of the information. For example two trees could have several subset trees in common, however their differences are not accounted for. Adding several nodes while keeping the same number of subset trees in one of the trees may yield the same kernel ‘dot product’. The same applies to most of the kernel methods above. More flexible kernels like Elastic kernels or partial tree kernels are even worse in that sense, they allows the identification of edges that our Vectorial Tree Distance (VTD) method presented here will not allow.
- Spectrum kernels^20^ identify trees by searching for q-grams on the trees, these are patterns of predefined structure, possibly even labeled^21^. Q-grams are identified without respect to their position in the tree. Our VTD is highly dependent on the distance from the root.
- Fisher kernel is based on the generative stochastic model from which trees are drawn. Therefore different trees having the same statistical parameters are considered the same. Our VTD distance measure yields a distance between two specific trees.
- Self-Organizing Map of trees^9,22^ uses non-supervised learning by competitive networks, and is effectively a clustering method. It identifies trees in the same cluster and thereafter uses kernel methods. In that sense it could measure the similarity of clusters, but not of trees.

To end this short review we mention some recent developments in phylogenetic tree distance measures:

In^23^ it was shown that one can cluster phylogenetic trees into meaningful groups using the spectral decomposition of the Laplacian matrices. Moreover, eigenvalues’ gap were identified with modes of division within a tree, such as rates of diversification. In^24^ a new tree distance was presented for rooted trees. For each pair of leaves, the most recent common ancestor (MRCA) was identified, then the distances of the MRCAs to the root was computed in two ways, either by summing the lengths of the edges or by summing the number of edges. Each tree was therefore given a weighted sum of two vectors. The distance between two trees was defined to be the Euclidean distance of the trees’ vectors. The new distance was successful in identifying different gene trees such as Ebolavirus. In^25^ a new measure of tree imbalance was suggested based on Suckin’s statistic. The authors compared the frequency of clades as computed under the Yule (the symmetrical) model with their empirical frequency in the data. This was done for clades of any size, yielding a vector of distances. The measure was tested on a simulated biased data against a null hypothesis of a Yule distribution. It was found that counting the number of ‘cherries’ is the most efficient way for detecting departure from the Yule model.

The algorithm presented here depends on the tree roots, and on the distance of the branches from the roots, therefore it is not allowed to swap nodes having different levels. This is the main reason why some of the above mentioned edit algorithms and their variants are different in principle^26^. Our algorithm resembles the tree alignment distance algorithm in^27^ restricted to the case where all labels are the same, trees are unordered and having bounded degree, a trivial cost function is defined on the labels, and the output is a scalar function; moreover we do not allow the insertion of internal ‘space’ nodes, only new boundary nodes are allowed. The time complexity of^27^ is of the order of $|T|^2$ where |*T*| is the number of vertices.

Our algorithm is more simplistic in the sense that it ignores all labels and therefore processes weaker information. Having no taxa we can permute sub-branches or use other symmetries and therefore some information such as in gene trees or species trees are lost. We can use the algorithm to cluster families of trees, different in their generating probability distribution, see “Clustering of simulated phylogenetic trees: comparing VTD to other methods” section, for example we can differentiate families of gene trees by the distance of their generating distributions from the Yule model. Having waved some of the information the algorithmic time complexity is reduced as we shall discuss later, see  “Discussion” section. We therefore trade information capacity with time complexity. We can get fast results on weak information. Note however that the algorithm was desiged as a general mathematical tool. Indeed its application for clustering phylogenetic trees is most natural, however we expect to find yet other fields of research in need for such a tool . In the following we assume each tree has a root (center), the existence of which is well known^28^, anyhow our version of the TDV package (see the discussion below for details) includes a simple function to find such a root.

Intuitively, given two trees and their corresponding centers, we will say that the trees are similar if there is a mapping taking one tree into the other, which is covariant with respect to distances from the center and with respect to descendancy. Covariance with respect to distance from the center means that for all *R*, a shell of radius *R* (from the center) of tree 1 is mapped into a shell of radius *R* of tree 2. Covariance with respect to descendancy means that whenever branch *a* of tree 1 is mapped into branch $a'$ of tree 2, the descendants of *a* are mapped into descendants of $a'$. If such a mapping exists we can say the two trees are similar, modulo a permutation or a naming of the branches.

**Figure 1:**
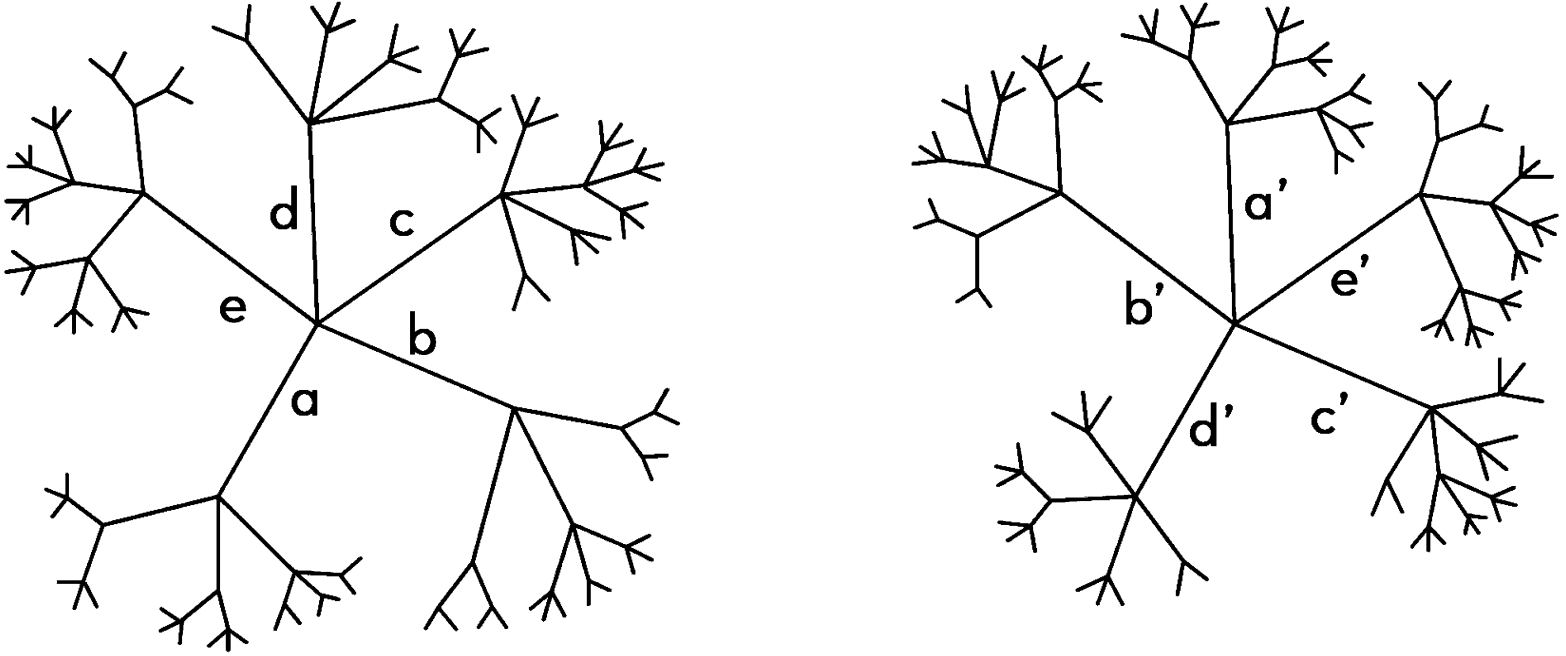
The above trees look very different; however if one maps the main branches (a, b, c, d, e) into (a′, b′, c′, d′, e′), it becomes apparent that the trees differ only by two extra leaves on the right hand tree, which is the difference between branches b and b’ at the 4th level from the center.

Below we develop the above mentioned mapping and measure the residual difference after applying it. Originally two trees might look different, and only following the suggested mapping one can recognize their similarity. For example, consider the following two simple trees (Fig. 1), which seem different. However, if one permutes the main branches mapping (a, b, c, d, e) into (a′, b′, c′, d′, e′) she will find that the only difference is two extra leaves on the right tree at the 4th level from the center, which is the difference between branch b and branch b′.

Having recursively T-aligned two trees (see detailed description in “The vectorial tree distance algorithm” section), we define the Vectorial Tree Distance as:

the vector whose entries are the differences in the number of branches at each level.

**Figure 2:**
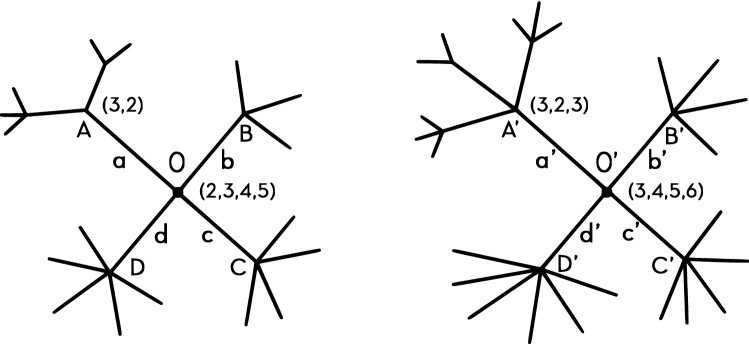
Simple example of trees’ alignment.

Here is the algorithm in a nutshell (a detailed description is given in “The vectorial tree distance algorithm” section). Suppose we are given two trees, each with its center point. We T-align the trees from their centers outwards, starting from their stumps. We attach an *n*-ary vector to each of the centers, where *n* is the number of branches in the stump, and each entry is the number of sub-branches, i.e. descendants of that branch, see Fig. 2. For example, we attach the vector (2,3,4,5) to node *O* since it has 4 branches: 2 sub-branches for node *A*, 3 for node *B*, 4 for node *C*, and 5 for node *D*. This ‘one step look ahead’ weighting method is similar to the one suggested by the ‘k-shell’ decomposition algorithm, see^29^ and references therein. The ‘look-ahead’ method is defined as follows: Let *O* be the root of a tree, and $V(O) = (n_1,...,n_k)$ be the weight vector of *O*, where *k* is the outgoing degree of *O*, and $n_k$ is the number of outgoing branches of the *k*th branch of *O* (we do not count the edge between *O* and its *k*th branch). Similarly we define the weight vector for any node, using the same direction, defined by the root. Next, we align the two stumps to minimize the $L_1$ difference of the vectors. This is the ‘weighted matching problem’^30^, and there can be several such matchings. This minimal $L_1$ difference will be the first entry of the VTD. Freezing this alignment we will force an alignment of the next level, i.e. their sub-branches. Thus if branch *a* in the first tree’s stump is paired with branch $a'$ in the second tree, then the descendants of *a* will be aligned with the descendants of $a'$. For the current level we use the same look-ahead method and attach *n*-ary vectors to each node at distance 1 from the center (for example nodes *A* and $A'$ in Fig. 2), where *n* is the number of sub-branches for the corresponding branch, and the entries are the number of sub-sub-branches for each sub-branch; for example, we attach the vector (3,2) to node *A* since it has 2 descending branches, one with 3 sub-branches, the other with 2 sub-branches. The set of all $L_1$ minimal distances at that level (the level of nodes *A*, *B*, *C* and *D*) defines an alignment of the sub-branches, and their sum will be the next entry of the VTD. This process is continued recursively. In case there is more than one minimal alignment at any level, the above process is continued with all those alignments in parallel.

Here is the definition of our ‘look-ahead’ method: Let *O* be the root of a tree. Let $V(O) = (n_1,...,n_k)$ be the weight vector of *O*, where *k* is the outgoing degree of *O*, and $n_k$ is the number of outgoing branches of the *k*th branch of *O*. Implicit in the definition is a direction from the root *O* outwards (in computing $n_k$ we do not count the edge between *O* and its *k*th branch). Similarly we define the weight vector for any node, using the same direction, defined by the root. We can use a simple Breadth First Search to find the nodes’ weights vector. When comparing two weight vectors, in case they have different lengths, we pad the shorter one by adding zeroes.

A remark concerning computational complexity. We assume the degree of each vertex in both trees is bounded by some integer *k*. Then for random trees the complexity of the VTD is *O*(*k*|*V*|) where |*V*| is the number of vertices (the maximal), see the discussion below, “Discussion” section.

In the next section we present some definitions and preliminaries. In “The vectorial tree distance algorithm” section we elaborate on the algorithm along with detailed examples. In “Clustering of simulated phylogenetic trees: comparing VTD to other methods” section we compare our algorithm to other known methods in the task of clustering sets of phylogenetic trees generated by the TreeSimGM simulator^31,32^. In Sect. “Clustering strains of fungi phylogenetic trees” we test the VTD on real data; phylogenetic trees extracted from two members of the fungi kingdom, mushrooms and mildews.

The Matlab code can be accessed via: https://gitlab.com/avner.priel/vectorial-tree-distance.

## The vectorial tree distance algorithm

We start with some definitions and notations to be used later.

### Definitions and preliminaries

#### Definition 1

** A Tree-alignment (T-alignment) of two trees:** Given two trees $Tr_1$ and $Tr_2$ and their corresponding centers $C_1$ and $C_2$, we will say that a mapping *Al* is a T-alignment of the trees if for every level *R* from both centers, *Al* maps the branches of one tree in that level to the branches of the other tree in the same level (with possible zero-padding into ghost branches), conditioned on the constrains that if branch *a* of tree $Tr_1$ is T-aligned with branch $a'$ of $Tr_2$, then all descendants of *a* will be aligned with all descendants of $a'$.

#### Definition 2

** Minimal T-Alignment:** Given two trees $Tr_1$ and $Tr_2$ and a T- alignment *Al* of the trees, we say that *Al* is minimal if for every level *R*, going over all possible T-alignments at level *R*, *Al* is such that the difference between the number of descendants of any two T-aligned branches, summed over all pairs of T-aligned branches in that level *R* is minimal.

#### Definition 3

** Vectorial Tree Distance:** Given two trees $Tr_1$ and $Tr_2$ and a minimal T-alignment *Al*, then the VTD of the two trees is the vector whose *R*-entry is the minimal difference given by the minimal T-alignment *Al* for the level *R*. We let *D* denotes the distance vector.

Note that the first two definitions above are recursive, namely, the constrains must be fulfilled for all levels *R*. At each level *R*, the sum of all minimal $L_1$ differences is the *R* entry of the distance vector.

In the following section, we elaborate on the algorithm. After describing the main steps of the procedure, we focus on the details via two specific examples.

### T-Alignment and comparison

Given a pair of trees and center nodes we follow the trees from these nodes outwards. At **level 0** we simply compare the number of edges of the stumps. The difference is the 0th entry of the distance vector. Suppose one stump has *m* edges the other stump has *k* edges, such that $m \ge k \ge 0$ (without loss of generatlity), then the **0-entry** of the distance vector is $m-k$. At **level 1**, there are several ways to T-align the *m* edges onto the *k* edges. We attach an *n*-ary vector to the center of each tree, where *n* is the number of branches in the corresponding stump, and each entry is the number of sub-branches, i.e. descendants of that branch, see examples below. The T-alignment problem is thus reduced to a ’weighted matching problem’^30^. We search for a T-alignment such that the $L_1$ norm distance between the two weight vectors is minimal. The minimal difference of weights will be the **1-entry** of the distance vector. It could be that more than one T-alignment has the same minimal difference; in that case, all such T-alignments are kept for the next step(s). At **level 2** we T-align the edges at distance 2 from the center, conditioned on the T-alignment(s) of the previous step. For that, we attach weight vectors to the nodes at level 1, looking ahead, the same way as above. At this stage we have several instances of the ’weighted matching problem’, and we sum all $L_1$ norm distances at that level. The **2-entry** of the distance vector will be the minimum over all such sums of $L_1$ distances, going over all good (minimal) T-alignments of the previous level. This process is recursively continues until all nodes are exhausted. The following examples elaborate on the first few steps of the procedure.

#### Example 1

**Figure 3:**
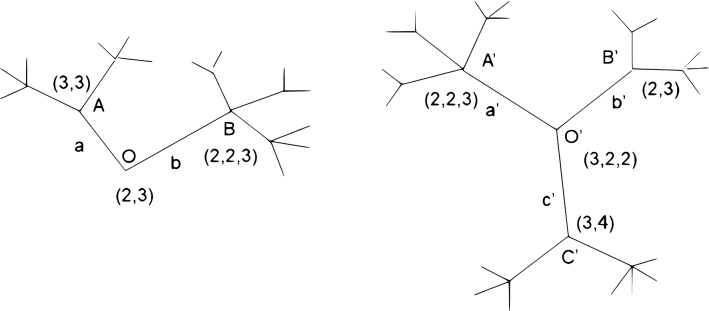
Detailed T-alignment of two trees.

**Figure 4:**
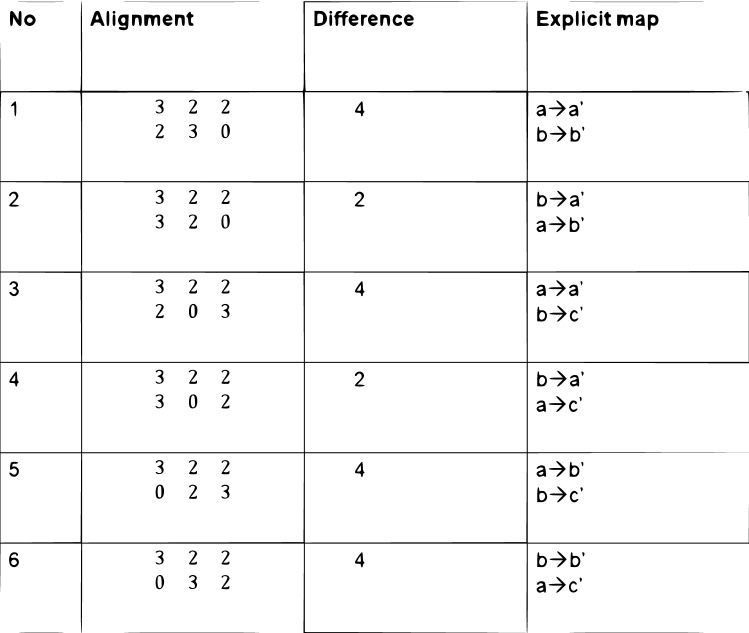
First level matching table.

Consider the trees in Fig. 3. The **0-entry** of the distance vector $D(0)=3-2=1$, which is the difference in the number of branches of the stumps. Attach the vector (2,3) to node *O*, corresponding to the two sub-branches stemming out of node *A* and 3 sub-branches stemming out of node *B*. Similarly we attach the vector (3,2,2) to node $O'$ of the second tree. We fix the right tree and map the branches *a* and *b* of the left tree into the branches $a'$ $b'$ and $c'$ of the right tree at level 1. There are 6 such mappings, each is denoted by a permutation of the weight vector (2,3,0) (while the vector (3,2,2) of the left tree fixed). The table in Fig. 4 show all possible mappings, where we zero-pad the vector at *O* i.e. $v(O) = (2,3,0)$, to allow permutations of two vectors with different sizes. The 3’rd column of the table includes the minimal $L_1$ differences, and the 4’th column gives the explicit mappings between the nodes. Observe that the 2’nd and 4’th mappings (see the third column of the table) both have $L_1$ minimal difference that equals 2. Therefore the 1’st entry of the distance vector $D(1)=2$, and there are 2 minimal T-alignments at that level. How can we tell which of the two T-alignments is preferred? For that we will have to take a look at the next level T-alignments.

At the next level T-alignments we first attach vectors to the nodes at distance 1 from the centers; for the first tree we set $v(A) = (3,3,0)$ and $v(B) = (2,2,3)$ , and for the second tree we set $v(A') = (2,2,3)$, $v(B') = (2,3,0)$ and $v(C') = (3,4)$. Next we T-align the branches of level 2 conditioned on the two minimal T-alignments from the previous step. If we follow T-alignment number 2 of the first level (see the table in Fig. 4), then *b* is mapped to $a'$ and we have to align the descendants of *b* with the descendants of $a'$. Therefore, we have to match $v(B)= (2,2,3)$ with $v(A')= (2,2,3)$ which has 2 possible minimal matchings (where we permute the sub-branches with two leaves) with both $L_1$ difference equals 0. Similarly, since *a* is mapped to $b'$ we have to match $v(A)= (3,3)$ with $v(B')=(2,3)$ which has 2 possible minimal matchings, and a minimal difference equals 1. To this $L_1$ difference at level 2 we have to add the number of (ghost) branches at level 2 which are the descendants of $c'$, i.e. 7. Therefore, if we follow the 2’nd T-alignment at level 1 we end with a sum of differences equals 0 + 1 + 7 = 8 at level 2, which is the sum of 3 $L_1$ differences. Alternatively, if we follow the 4’th T-alignment at level 1 (4’th line on the table), we have to map *b* into $a'$, and therefore we have to match $v(B)= (2,2,3)$ with $v(A') =(2,2,3)$ which again has 2 possible minimal matchings and difference equals 0. Additionally, we map *a* into $c'$ and therefore match $v(A)= (3,3)$ with $v(C') = (3,4)$, which has 2 possible minimal matchings with difference equals 1. To this $L_1$ difference we have to add the number of branches at level 2 which are the descendant of $b'$, i.e. 5. Therefore if we follow the 4’th T-alignment at level 1 we end with a difference of 0 + 1 + 5 = 6. The 4’th T-alignment at the first level is therefore better and hence the 2’nd entry of the distance vector $D(2)=6$. To summarize, the first 3 entries of the distance vector *D* are: $D = (1,2,6)$

**Figure 5:**
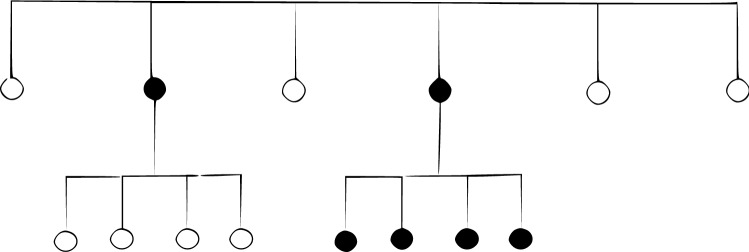
The set of possible mappings.

In Fig. 5 we present the procedure describing the set of all mappings for the above example. An open circle denotes a T- alignment and a closed one denotes a T- alignment with minimal distance value. At the first level we had 6 possible T-alignments (see the table in Fig. 4), 2 were minimal with $L_1$ distance equals 2, and the other 4 with $L_1$ distance equals 4. At the second level we extend only the minimal T-alignments of the first level. To each minimal T-alignment of the first level we had 4 sub T-alignments. The 2’nd T-alignment of the first level had 4 sub T-alignments with $L_1$ distance 8, whereas the 4’th T-alignment of the first level had 4 sub T-alignments with $L_1$ distance 6.

The following example is more complex, however we will not go into too much details, having in mind the previous example.

#### Example 2

In this example (see Fig. 2) the distance at **level 0** is 0 since both stumps have 4 branches. Next we attach the weight vector (2,3,4,5) to the center node *O*, and the weight vector (3,4,5,6) to the center node $O'$ of the second tree. We now look at the set of all matches between the two weight vectors, having minimal $L_1$ sum. An optimal match would be to T-align *a* with $a'$, *b* with $b'$, *c* with $c'$, and *d* with $d'$. This would T-align the weights (2,3,4,5) with (3,4,5,6) having an overall distance equals 4. Therefore the first level distance coefficient is 4.

If we T-aligned *a* with $a'$ at the first level we will have to T- align the descendants of *a* with the descendants of $a'$. We attach a 2-tuple weight vector to *A*, (3,2), and a 3-tuple weight vector to $A'$, (3,2,3), and compute the minimal T-alignment between the two weight vectors. One of the branches of $a'$ will be left non T-aligned (ghost) and all the descendants of that branch will add a corresponding difference at each of the next levels. Similarly we attach weight vectors to *B* and $B'$, *C* and $C'$, *D* and $D'$. For each of the above pairs we compute the minimal T-alignment. Finally, we sum all the above minimal $L_1$ differences to obtain the entry for the distance vector. In case both weight vectors have 0 entries (as in this example for *B* and $B'$, *C* and $C'$, and *D* and $D'$), we can pick any T-alignment and the $L_1$ difference will be 0.

At level 1 we could T-align the branches *a* with $d'$, *b* with $a'$, *c* with $b'$, and *d* with $c'$, i.e. (2,3,4,5) with (6,3,4,5), where the minimal $L_1$ distance also equals 4 as above. If we map *a* to $d'$ we should T-align the descendants of *a* with the descendants of $d'$, therefore we should look at the 6-tuple weight vector for $d'$, and find its best match with the 2-tuple vector for *a*. We should also find the other minimal matches between the 3-tuple of *b* and the 3-tuple of $a'$, the 4-tuple of *c* and the 4-tuple of $b'$, and the 5-tuple of *d* and the 5-tuple of $c'$.

We will now compare the sum of $L_1$ differences of the 2’nd level we computed, conditioned on the first level T-alignment of (2,3,4,5) with (3,4,5,6), with the alternative sum of $L_1$ differences of the 2’nd level conditioned on the first level T-alignment of (2,3,4,5) with (6,3,4,5). In general, we will minimize the sum of $L_1$ differences of the 2’nd level going over all minimal T-alignments of the first level. It is easy to see that the VTD distance is (0,4,3). We leave the rest of the details to the reader.

Let us now provide the general formulation of the process in terms of ‘Dynamic-Programming’:

Let $$\overrightarrow{X} = (x_1,...,x_k)$$, $$\overrightarrow{Y} = (y_1,...,y_k)$$,

1 $$\begin{aligned} L_1(\overrightarrow{X},\overrightarrow{Y}) = \sum _{i=1}^k|x_i-y_i|; \end{aligned}$$

define

2 $$\begin{aligned} M (\overrightarrow{X},\overrightarrow{Y}) = min_{\sigma \in Sym(k)}L_1 (\sigma (\overrightarrow{X}),\overrightarrow{Y}) \end{aligned}$$

where *Sym*(*k*) is the Symmetric group on *k* elements. Let

3 $$\begin{aligned} M_{\sigma }(\overrightarrow{X},\overrightarrow{Y}) = arg_{\sigma } ( min_{\sigma \in Sym(k)}L_1 (\sigma (\overrightarrow{X}),\overrightarrow{Y})) \end{aligned}$$

denote the set of permutations in *Sym*(*k*) satisfying the above minimum. In case the two vectors are not of the same length we pad the shorter one with zeroes.

Let $D= D_{(O,O')}$ be the tree distance vector computed from the two centers outwards; *O* for tree 1, $O'$ for tree 2. Given a root *O*, let $V(O) = (n_1,...,n_k)$ be the weight vector for node *O*, where *k* is the degree of *O* and $n_k$ the number of sub-branches of *O* that are branches of the *k*th branch of *O*. Let $V_k(O)$ denotes the *k*th weight $n_k$ and $V^k(O)$ the corresponding node. Let $D^1_{V(O)^k,V(O')^j}$ be the tree distance vector for the two subtrees stemming outwards of vertices $V(O)^k$ and $V(O')^j$ each computed from its first coordinate outwards. Let *z* be a predetermined length of the measurement vector D. The vectorial tree distance can be computed by the following recursive process:

The first coordinate (level) of $D= D_{(O,O')}$ satisfies:

4 $$\begin{aligned} D_{(O,O')} (1)= M(V(O),V(O')) \end{aligned}$$

and the (2,..., z) coordinates (levels) satisfy:

5 $$\begin{aligned}&D_{(O,O')} (2,...,z)= \nonumber \\&=\mathbf{lexmin} _{\sigma \in M_{\sigma }(V(O),V(O'))} \bigg \{ D^1_{V(O)^1,V(O')^{\sigma (1)}} + \nonumber \\&\quad+ D^1_{V(O)^2,V(O')^{\sigma (2)}} \nonumber \\&\quad...+ D^1_{V(O)^k,V(O')^{\sigma (k)}} \bigg \} \end{aligned}$$

Namely, to compute the distance vector from any two root points, we look-ahead to the neighboring vertices, having computed the distance vectors for the corresponding pairs of sub-trees (paired by one minimal T-alignment of the root branches), we sum the vectors point-wise. We now compute the minimal sum under the lexicographic order, going over all minimal T-alignments of the root branches. To compute $D^1_{V(O)^i,V(O')^{\sigma (i)}}$ we need to re-use the above formula with $V(O)^i$ and $V(O')^{\sigma (i)}$ as the new roots. The zero coordinate of D is the difference of the root's degrees.

## Clustering of simulated phylogenetic trees: comparing VTD to other methods

In this section we demonstrate our VTD measure on the problem of clustering families of phylogenetic trees generated by the TreeSimGM package^31,32^. “TreeSimGM,” is an R-package simulation tool for generating stochastic phylogenetic trees under a general Bellman and Harris model^33^. The package allows the user to specify any desired probability distribution for the waiting times until speciation and extinction. Trees generated by the TreeSimGM have basic and simple parameters and can be considered as representing possible natural trees. We also compared our VTD with known tree distance measures; Robinson Foulds^34^, SPR distance^35^, KF distance^36^, and path distance^37^. We took 3 families of 60 trees each. On each family we tested a different property. We used k-means algorithms (k = 2) on the distance matrices to cluster each family according to the property tested. For the VTD measure we built a k-means algorithm anew, denoted vectorial k-means (VKM). This variant defers from standard k-means in the following aspects. First, the cluster-center in the VKM is a representative tree chosen to be the closest to all other members (trees) of the cluster. Although this ’closeness‘ measure can be obtained in many ways, for our analysis we simply used the root-mean-square of the VTD between pairs of trees. Second, the association stage, i.e. tree-to-cluster assignment is also done in the vector sense, i.e., the cluster chosen for each tree is obtained by calculating the VTD to any of the clusters’ representatives and selecting the one with the minimal root mean square. This process is repeated iteratively.

Here are the details followed by a table of the results:

a) Symmetric trees versus asymmetric trees—A set of 60 trees was generated. A sub-set of 30 was generated by a symmetric process, the other by a-symmetric generator. We used the same taxa 10 for all trees to allow the comparison with classical tree distance measures. In all trees a Weibull(3,0.1) was used as the waitsp (waiting time for speciation) distribution and an exp(0) as the waitext (waiting time for extinction) distribution.

b) Hierarchical versus non-hierarchical trees—A set of 60 trees was generated. A sub-set of 30 was generated by an hierarchical process, the other by a non-hierarchical generator. To construct hierarchical trees we used Weibull(4,0.1) as the waitesp distribution, and Weibull(1,0.1) for the non-hierarchical case. Otherwise all trees had taxa 15 and waitext distribution of exp(0). All trees were asymmetrically generated.

c) Trees of different taxa - in this case the classical measures are harder to use for measuring the distance between trees. We demonstrated the fact that the VTD measure can easily separate between different speciation parameters. We compared trees having waitsp distribution exp(0.5), with trees having waitsp of exp(0.2). Otherwise all trees had waitext distribution of exp(0.3). All trees were asymmetrically generated with the same age parameter, sim.age equals 10.

**Figure 6:**
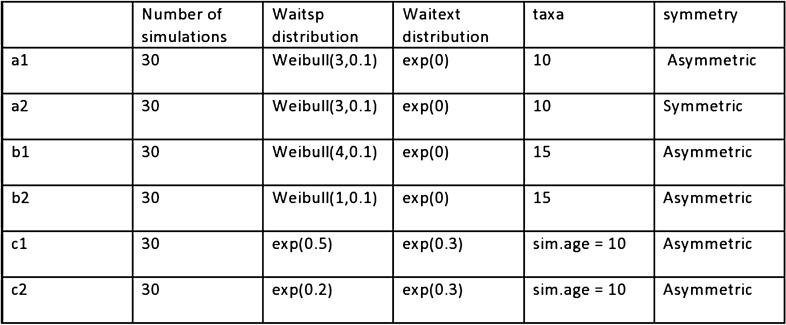
Details of TreeSimGM simulations.

**Figure 7:**
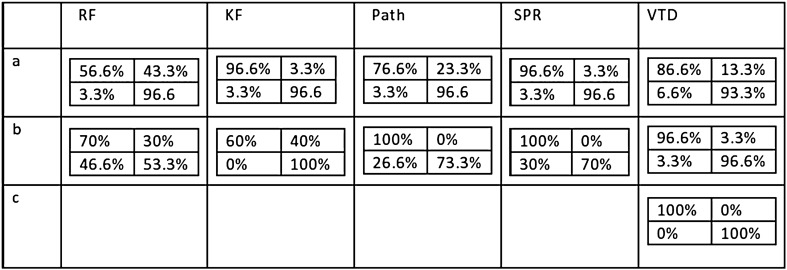
Confusion matrices for the above experiments.

The table in Fig. 6 summarizes the details of the experiments. The table in Fig. 7 summarizes the details of the results. In separating symmetric versus asymmetric trees the VTD measure showed better results than the RF and path distance measures. In separating hierarchical versus non-hierarchical trees the VTD measure showed better results than all the above methods. The VTD distance could also well separate trees having different waitsp (exponential) distributions with different taxa.

## Clustering strains of fungi phylogenetic trees

**Figure 8:**
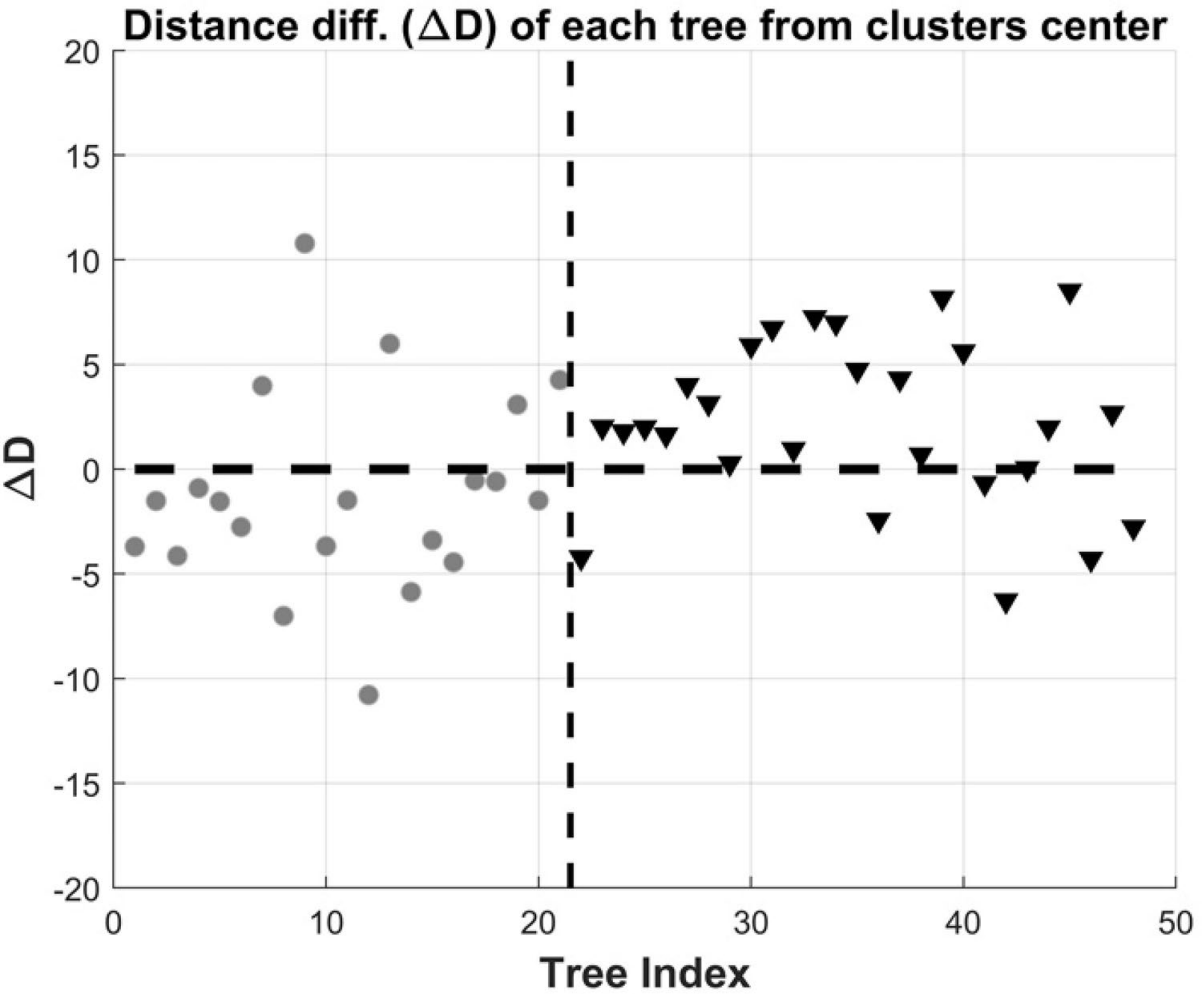
Clustering mushrooms versus mildews phylogenetic trees. First/second cluster refers to mushroom/mildew respectively, hence the majority of samples 1-21 have negative difference values, whereas the majority of samples 22-48 have positive values (i.e., they are closer to the second cluster).

To demonstrate the power of the VTD measure, we analyzed two sets of trees derived from two members of the fungi kingdom, mushrooms and mildew. The data is taken from TreeBASE^38,39^, and consists of sets of species trees. The set consists of 21 (res. 27) phylogenetic trees of mushrooms (resp. mildew). Initially we combine the two sets and create a set of 48 trees. Next, we calculate the VTD for each pair of trees, hence obtaining a 3D matrix of dimension [N x N x L] where N is the total number of trees and L is the depth (level) for which the distance is calculated. We apply the vectorial k-means algorithm (described in "Clustering of simulated phylogenetic trees: comparing VTD to other methods" section) and obtain the centers of the two clusters (assuming k = 2). We also apply the k-medoids algorithm^40^ with similar results. In order to use a scalar distance value for each pair (rather than a vector), we simply sum the distance values of levels 2–5. Figure 8 depicts the results of this partition. Once the algorithm converges to the k = 2 trees that are the centers (medoids), we pick the distances of all trees from these centers. The x-axis is simply the running index of the tree, where the circles (1–21) belong to the mushroom set, the triangles (22–48) to the mildew set. The y-axis is the difference of distances of each tree *i* from the two centers, $(D_{i,1} - D_{i,2})$. Hence, negative value means the tree is closer to the first center. The horizontal black dashed line is at D = 0 for reference, as well as the vertical dashed line separating the two sets of trees. As can be seen, 16/21 of the trees in the first set (mushroom) are closer to the first center, and 21/27 of the trees in the second set (mildew) are closer to the second center. The confusion matrix representing the results is [0.7619 0.2380; 0.2222 0.7777]. The results clearly show that it’s possible to differentiate between sets of phylogenetic trees generated from different sources or distributions.

## Discussion

In this manuscript we present a new vector distance measure for a pair of trees, denoted VTD. The distance vector is calculated following a T-alignment process of the trees, applied from a given pair of center points outwards. We describe the algorithm in details.

To compare the VTD to other well known tree distance measures we simulated several families of phylogenetic trees using the TreeSimGM R-package. In separating symmetric versus asymmetric trees the VTD measure showed better results than the RF or path distance measure, and in separating hierarchical versus non-hierarchical trees it was found better than the RF, KF, Path, or SPR distance measure.

Some remarks concerning computational complexity. We assume the degree of each vertex in both trees is bounded by some integer *k*. At each vertex we compute a weighted matching on two *k*-dimensional integer vectors. The ‘weighted matching problem’ is known to have polynomial complexity (in *k*)^30^. Furthermore, finding **all** minimal solutions to the weighted matching problem is also polynomial (in *k*)^41^. Denote by |*M*(*k*)| the complexity of finding all minimal matchings of two *k* dimensional integer vectors, let $\#M(k)$ be the number of such minimal alignments. Consider now the process of T-alignment, going from the center outwards. At each level, and for each T-alignment we compute the matching between the nodes aligned. Therefore the complexity will be the product of |*M*(*k*)| by the number of vertices at that level by the number of T-alignments at that level. Multiple equivalent T-alignments at any level could have been originated at a previous level, or could have been originated at that level by equivalent mathcings. For random trees multiple T-alignments from both sources are rare. We can therefore characterize the tree of T-alignments described in Fig. 5. Having all that in mind it is easy to see that for random trees the complexity of the VTD is *O*(|(*M*(*k*)| |*V*|) where |*V*| is the number of vertices (maximal). The exact proof is left for future research.

Most algorithms for the alignment of pairs of trees have higher time complexity of the order of $O(|V_1||V_2|)$ (see Table 1 in^2^), however these algorithms align trees with labels. Our algorithm is more simplistic in the sense that it ignores all labels and therefore processes weaker information, however its time complexity is lower.

Our algorithm can be used to gain some insights in several areas, such as game theory, decision-making processes, genetics, communication networks and more. For example in the area of decision making, assuming it is possible to map the decision processes into trees. Then one can define a distance measure between two such processes, each may occur in different organizations, or situations. This could also be applied in crisis management cases where complex scenarios can be mapped into a corresponding tree and further analyzed against other scenarios or use cases.

In immunology, currently it is possible to obtain the repertoire sequencing of various immune cells, e.g. B/C cells^42,43^ from peripheral blood. This means that different subjects, or even the same subject at different time points, exhibit different repertoires. One of the major hurdles is to infer useful insights from this vast information. We suggest that applying the vector distance measure, it is possible to compare repertoires after they have been mapped to their network representation.

## References

[1] Wilson RC, Zhu P (2008). A study of graph spectra for comparing graphs and trees. J. Pattern Recogn..

[2] Bille P (2005). A survey on the tree edit distance and related problems. Theoret. Comput. Sci..

[3] Zhang, M., Jiang, H., Aw ,A. T., Sun, J., Li, S. & Tan C.L. A tree-to-tree alignment-based model for statistical machine translation. In *MT-Summit-07* 535–542 (2007).

[4] Zhang, M., Jiang, H., Aw, A. T., Sun, J., Li, S. & Tan, C. L. A tree sequence alignment-based tree-to-tree translation model. In *ACL-08: HLT-46th Annual Meeting of the Association for Computational Linguistics: Human Language Technologies, Proceedings of the Conference* 559–567 (2008).

[5] Robinson DF, Foulds LR (1981). Comparison of phylogenetic trees. Math. Biosci..

[6] Scornavacca C, Zickman F, Huson DH (2011). Tanglegrams for rooted phylogenetic trees and networks. Bioinformatics.

[7] Nye TMW, Lio P, Gilks WR (2006). A novel algorithm and web based tool for comparing two alternative phylogenetic trees. Bioinformatics.

[8] Robinson O, Dylus D, Dessimoz C (2016). Phylo.io: Interactive viewing and comparison of large phylogenetic trees on the web. Mol. Bio. Evol..

[9] San Martino, G. D. *Kernel Methods for Tree Structured Data*. Phd. Thesis, University di Bologna, Padova (2009)

[10] Kuboyama, T. *Matching and Learning in Trees*. Phd. Thesis, University of Tokyo (2007)

[11] Theodoridis S, Koutroumbas K (2009). Pattern Recognition.

[12] Vishwanathan, S. & Smola, A. J. Fast kernels on strings and trees. In *Proceedings on Neural Information Processing Systems* (2002).

[13] Collins, M. & Duffy, N. New ranking algorithms for parsing and tagging: Kernls over discrete structures, and the voted perceptron. In *ACL02* (2002).

[14] Moschitti, A. Making tree kernels practical for natural language learning. In *11th Conference of the European Chapter of the Association for Computational Linguistics* (2006).

[15] Rieck, K., Brefeld, U., Kruger, T. *Approximate Kernels for Trees*. Technical report, Fraunhofer Publica (2008).

[16] Moschitti, A. Efficient convolution kernels for dependency and constituent syntactic trees. In *ECML* (2006).

[17] Kashima, H. & Koyanagi, T. Kernels for semi-structured data. In *ICML* (2002).

[18] Zhang, M., et al. A grammar-driven convolution tree kernel for semantic role classification. In *ACL * (2007).

[19] Bloehdorn, S. & Moschitti, A. Structure and semantic for expressive text kernels. In *CIKM 07: Proceedings of the 16th ACM Conference on Conference on Information and Knowledge Management, N.Y. 2007* (ACM, 2007).

[20] Kuboyama, T., Hirata, K., Kashima, H., Aoki-Kinoshita, K. F. & Yasuda, H. A spectrum tree kernel. *Inf. Media Technolog.* **2**(1) (2007).

[21] Yamanishi, Y. Glycan classification with tree kernels. *Bioinformatics* **23**(10) (2010). 10.1093/bioinformatics/btm090 17344232

[22] Peura, M. The self-organizing map of trees. *Neural Process. Lett.* **8** (1998).

[23] Lewitus, E. & Morlon, H. Characterizing and comparing phylogenies from their Laplacian spectrum. *Syst. Biol.* **65**(3) (2016). 10.1093/sysbio/syv116 26658901

[24] Kendall, M. & Colijn, C. Mapping phylogenetic trees to reveal distinct patterns of evolution. *Mol. Biol. Evol.* **33**(10) (2016). 10.1093/molbev/msw124 PMC5026250 27343287

[25] Blum, M. G. B. & Francois, O. On statistical tests of phylogenetic tree imbalance: The Sackin and other indices revisited. *Math. Biosci.* **195** (2005). 10.1016/j.mbs.2005.03.003 15893336

[26] Gusfield D (1997). Algorithms on Strings, Trees, and Sequences.

[27] Jiang, T., Wang, L. & Zhang, K. Alignment of trees—an alternative to tree edit. *Theor. Comput. Sci.* **143**, 137–148 (1995).

[28] Hedetniemi SM, Cockayne EJ, Hedetniemi ST (1981). Linear algorithms for finding the Jordan center and path center of a tree. Transp. Sci..

[29] Carmi S, Havlin S, Kirkpatrick S, Shavitt Y, Shir E (2007). A model of Internet topology using k-shell decomposition. PNAS.

[30] Lawler EL (1976). Combinatorial Optimization: Networks and Metroids.

[31] Hagen O., & Stadler T. TreeSimGM: Simulating phylogenetic trees under general Bellman Harris models with lineage-specific shifts of speciation and extinction in R. *Methods Ecol. Evol.* **9**(3), 754–760 (2018). 10.1111/2041-210X.12917 PMC5993341 29938014

[32] Hagen O, Hartmann K, Steel M, Stadler T (2015). Age-dependent speciation can explain the shape of empirical trees. Syst. Biol..

[33] Bellman R, Harris TE (1948). On the theory of age-dependent stochastic branching processes. Proc. Nat. Acad. Sci. U.S.A..

[34] Robinson DF, Foulds LR (1981). Comparison of phylogenetic trees. Math. Biosci..

[35] Hein J, Wang L, Zhang K (1996). On the complexity of comparing evolutionary trees. Discrete Appl. Math..

[36] Kuhner MK, Felsenstein J (1994). A simulation comparison of phylogeny algorithms under equal and unequal evolutionary rates. Mol. Biol. Evol..

[37] Steel MA, Penny P (1993). Distributions of tree comparison metrics—some new results. Syst. Biol..

[38] Piel, W. H., Chan, L., Dominus, M. J., Ruan, J., Vos, R. A. & Tannen, V. TreeBASE v.2: A database of phylogenetic knowledge in e-BioSphere (2009).

[39] Vos, R. A., *et al.* NeXML: Rich, extensible, and verifiable representation of comparative data and metadata. *Syst. Biol.* **61**(4), 675–689 (2012). 10.1093/sysbio/sys025 PMC3376374 22357728

[40] Kaufman L, Rousseeuw PJ (2005). Finding Groups in Data: An Introduction to Cluster Analysis.

[41] Fukuda K, Matsui T (1992). Finding all minimum cost perfect matchings in bipartite graphs. Networks.

[42] Yermanos, A., *et al.* Comparison of methods for phylogenetic B-cell lineage inference using time-resolved antibody repertoire simulations. *Bioinformatics* **33**(24), 3938-3946 (2017). 10.1093/bioinformatics/btx533 28968873

[43] Priel, A., Gordin, M., Philip, H., Zilberberg, A., & Efroni S. Network representation of T-Cell repertoire—a novel tool to analyze immune response to cancer formation. *Front. Immunol.* **9**. ISSN: 1664-3224. 10.3389/fimmu.2018.02913 (2018). 10.3389/fimmu.2018.02913 PMC6297828 30619277

